# Antioxidant Supplementation Reduces *In Vitro* Oxidant Generation in Neonatal Total Parenteral Nutrition Solutions

**DOI:** 10.1016/j.cdnut.2026.107694

**Published:** 2026-04-15

**Authors:** Kandeepan Karthigesu, Robert F Bertolo, Robert J Brown

**Affiliations:** Department of Biochemistry, Memorial University of Newfoundland, St. John’s, NL, Canada

**Keywords:** total parenteral nutrition, antioxidants, newborns, peroxidation, prooxidants

## Abstract

**Background:**

Newborns with gastrointestinal anomalies or premature birth often require lifesaving nutritional support in the form of total parenteral nutrition (TPN). However, TPN administration causes several complications in newborns. Several studies have suggested that it could be due to oxidant generation in the TPN admixture because of various factors.

**Objectives:**

This study aimed to reduce the peroxidation of the commercially available TPN for newborns by increasing concentrations of antioxidants, decreasing concentrations of prooxidants, and optimizing environmental conditions, leading to the development of a new admixture with lower oxidant load.

**Methods:**

Standard all-in-one (AIO)-TPN was used as a control diet and various antioxidants or prooxidants were supplemented above standard concentrations to determine effects on peroxide concentrations. TPNs were also exposed to light to determine environmental effects on oxidant load.

**Results:**

Peroxide concentrations in the AIO-TPN significantly increased with higher light intensity. Compared with standard AIO-TPN, AIO-TPN with a combination of L-ascorbic acid (at 2.27 mM), DL-α-tocopherol acetate (at 124.1 μM), glutathione (at 40 μM), copper (at 4.2 μM), and zinc (at 30.6 μM) in the AIO-TPN had significantly lower peroxide concentrations. The increasing combinations of vitamins C and E showed an additive effect on lowering the peroxide concentrations, whereas various combinations of copper and zinc alone did not change peroxide concentrations.

**Conclusions:**

Peroxide concentrations were significantly increased when TPN was exposed to light, but optimization using a specific combination of antioxidants significantly reduced the degree of peroxidation. Overall, this study gives novel insight into a promising strategy to develop a new TPN formula for newborns, particularly preterm infants, to decrease *in vitro* oxidant load.

## Introduction

Total parenteral nutrition (TPN), a lifesaving therapeutic solution, is administered to newborns in neonatal intensive care units (NICU) globally. It is indicated for newborns born with intestinal atresia, pyloric stenosis, biliary atresia, Hirschsprung disease, short bowel syndrome, or preterm <32 wk and/or <1500 g [1]. The WHO reported that 13.4 million infants were born prematurely in 2020. Not surprisingly, ∼900,000 newborns died in 2019 because of complications associated with prematurity [2,3]. In Canada, an estimated 8% of newborns are born prematurely at 37 wk or less [4]. Over the last 18 y, the proportion of low birth weight infants (<2.5 kg) increased from 5.9% in 2003 to 7.0% in 2022 [5]. Furthermore, 1 in 7 newborns in the United Kingdom were admitted to the NICU [6]. Newborns admitted to the NICU were hospitalized for a median of 4 d in term infants, to 92 d in infants <27 weeks of age [7]. However, an infant may stay even longer depending on the gastrointestinal conditions, surgery, and metabolic disorders. Such infants often require intervention with a balanced nutrient supplementation in the form of TPN to fulfill the daily requirement and for catch-up growth.

Although TPN is often unavoidable, nutrient solutions to such infants have numerous benefits, including catch-up growth, enhanced immune system, and provision of all essential nutrients; however, TPN feeding also causes many adverse effects. For instance, prolonged feeding with TPN can lead to bronchopulmonary dysplasia, liver disease and cholestasis, necrotizing enterocolitis, and gut atrophy [8]. These detrimental effects may be due to an increased delivery of oxidants from the TPN. Several studies have demonstrated that TPN generates oxidants under various conditions, including the mixing of elemental nutrients and lipid together, light exposure, and oxygenation.

Newborns are at a higher risk of oxidative stress because of the exposure of oxidants at an early growing period, from various endogenous and exogenous sources [9]. For instance, numerous endogenous sources of oxidative stress are from birth trauma, reperfusion injury from hypoxia, oxygen therapy, phototherapy, mechanical ventilation, infection, and inflammation [10,11]. Exogenous sources of peroxides are from diets consumed by or, in the case of TPN, infused to newborns. Besides diet, if oxygen therapy was used during the neonatal period or diet was exposed to light, those can be additional sources of oxidant load exacerbating oxidative stress [12,13]. The exposure of TPN to ambient light, daylight, or to light during phototherapy of a neonate induces the generation of peroxides. The oxidation of lipid emulsions is particularly exacerbated when exposed to ambient light or phototherapy in a clinical setting. In particular, preterm infants in the NICU often require TPN in the first weeks of life or longer, when they are voluntarily exposed to natural or artificial light to prevent neonatal jaundice [14]. Laborie et al. [[15], [16]] found that the peroxide concentration in light-exposed (LE) TPN was between 190 and 300 μM, compared with 60 and 130 μM when TPN was protected from light. Fat-free-TPN (FF-TPN), including amino acids and vitamin mixtures without lipids, can also be contaminated with H_2_O_2_ after mixing with LE riboflavin [17,18].

Despite *in vivo* antioxidant systems such as vitamin E, vitamin C, superoxide dismutase, catalase, and glutathione [19], which play major roles in reducing oxidative stress, neonates are even more prone to oxidative damage because antioxidant systems in newborns are immature, especially in preterm infants. Moreover, premature neonates, to whom TPN is prescribed more often, are more likely to be exposed to high amounts of peroxides. In addition, neonates just after birth are exposed to a relatively hyperoxic environment because of high oxygen bioavailability that causes the generation of free radicals [20]. Moreover, the antioxidant systems that remove these peroxides work at even lower capacity in premature neonates [[21], [22], [23]]. There are also antioxidant systems that depend on sufficient dietary intake of precursors. For example, glutathione is the most abundant nonprotein thiol-containing intracellular antioxidant. The biosynthesis of glutathione depends on sufficient dietary precursors, including cysteine [24]. However, the addition of cysteine to TPN is limited because of various reasons, including oxidation [25]. Hence, incorporating glutathione, in the form of glutathione disulfide (GSSG) into TPN prevented the oxidized redox potential of glutathione (high GSSG), activation of caspase-3 (apoptosis marker), and loss of alveoli [26]. Therefore, exogenous antioxidants and precursors for the synthesis of antioxidants should be supplied via TPN in neonates.

The purpose of this study was to determine the optimal concentrations of select antioxidants and prooxidant micronutrients for TPN that minimize oxidant development. Specifically, the antioxidants vitamins C and E, selenium, and glutathione were examined, along with the prooxidant trace elements copper and zinc. In addition, effects of exposure to the newly designed TPN were examined.

## Methods

### Chemicals and supplies

Ammonium ferrous sulfate (cat. #FX0245-1), xylenol orange (cat. #3618-43-7), DL-α-tocopherol acetate (cat. #47786), zinc sulfate (cat. #Z0251), copper sulfate (cat. #C8027), selenium dioxide (cat. #200107), glutathione (GSSG) (cat. #G4376), H_2_O_2_ 30% (*w/w*) in H_2_O (cat. #H1009), and *tert*-butyl hydroperoxide 70 wt. % in H_2_O (cat. #458139) were purchased from Millipore Sigma. L-ascorbic acid (>99%) (cat. #A1561322) was purchased from Thermo Scientific. Butylated hydroxytoluene (BHT) (cat. #ICN10116290) was purchased from Fisher Scientific.

To prepare the TPN admixture, 70% *w/v* dextrose (cat. #2B0296H), 10% *w/v* amino acids (cat. #FCA3CG133C29D); Baxter exactaMix 500 mL ethylene-vinyl acetate (EVA) bags (cat. #E3005OD), and intravenous infusion tubes (cat. #2C8401) from Baxter Corporation; Multi-12/K_1_ Pediatric vitamins (cat. #106909), MICRO+6 pediatric trace elements (cat. #106931); 20% magnesium sulfate (200 mg/mL) (cat. #00392618) from Sandoz Canada Inc; 32.8% sodium acetate from Omega Laboratories Limited (cat. #02181746); potassium phosphate (phosphorus 3 mmol/mL and potassium 4.4 mEq/mL) (cat. #C860539); 10% calcium gluconate (100 mg/mL) (cat. #C360019); sterile water; 1000 units/mL heparin sodium (cat. #C504013); and 20% *w/v* lipid injectable emulsion (SMOFlipid) (cat. #830820610) from Fresenius Kabi were purchased from the Janeway Children’s Hospital Pharmacy.

### Preparation of TPN solutions

FF-TPN and all-in-one (AIO)-TPN were prepared by mixing the above-mentioned ingredients, similar to those used in clinical settings. Briefly, 10% amino acid solution (Equivalent to 4 g protein/kg/d) (Supplemental Table 1) was first added into an exactaMix 500 mL EVA bag under sterile conditions. Subsequently, 70% dextrose (equivalent to 17 g carbohydrate/kg/d), MICRO+6 pediatric trace elements (Supplemental Table 2), electrolytes and multivitamin preparation (Supplemental Tables 3 and 4) were added. After preparation of FF-TPN (137.5 mL), the bag was wrapped with aluminum foil immediately. Finally, to make AIO-TPN commercially available, 20% SMOFlipid (equivalent to 2.5 g lipid/kg/d) was added to the FF-TPN at the ratio of 1:11, just before the experiment commenced. The pH of the solution was tested, and it was maintained within 5.6–6.7, as per previously reported studies [27,28]. The FF-TPN, SMOFlipid, and AIO-TPN solutions were all prepared under sterile conditions within a biosafety cabinet-II, with minimum light exposure (Lux 5-10).

### Measurement of light intensity

Light intensity was measured using a Traceable Dual-Display Light Meter (cat. #06-662-64, Fisher Scientific). For the light source, Ultra definition dimmable LED A19 E26 60W equivalent bright white light bulb (cat. #1001663118) (Philips) was used. The light intensity was adjusted from 0, 250, 500, 1000, 1500, and 3000 Lux to mimic the night and day NICU light and phototherapy light conditions.

### Peroxide concentrations of vitamins C– and/or E–added TPN samples

SMOFlipid was mixed with L-ascorbic acid to prepare 0 (baseline concentration), 5, 10, 20, 40, and 80 μM concentrations, whereas DL-α-tocopherol acetate concentration in commercially available SMOFlipid was 20 mg/100 mL (or 5 μM) (baseline). To prepare 10, 20, and 40 μM concentrations, DL-α-tocopherol acetate was added to the SMOFlipid.

The baseline vitamin C content in 150 mL AIO-TPN for newborns was 24 mg (or 0.91 mM). To such solution, 12, 24, 36, and 48 mg L-ascorbic acid was added to prepare 36 mg (1.36 mM), 48 mg (1.81 mM), 60 mg (2.27 mM), and 72 mg (2.73 mM) amounts. The pH was monitored after adding vitamin C to the SMOFlipid or AIO-TPN.

The baseline amount of DL-α-tocopherol found in the AIO-TPN was 4.6 mg/150 mL (or 64.87 μM). To such solution, 2.1, 4.2, and 6.3 mg DL-α-tocopherol acetate was added to prepare 6.7 mg (94.48 μM), 8.8 mg (124.10 μM), and 10.9 mg (153.7 μM).

Combinations of vitamin C at 24 mg (0.91 mM) (baseline), 36 mg (1.36 mM), 48 mg (1.81 mM), 60 mg (2.27 mM), and 72 mg (2.73 mM) and vitamin E at 4.6 mg (64.87 μM) (baseline), 6.7 mg (94.48 μM), 8.8 mg (124.10 μM), and 10.9 mg (153.7 μM) were used for *in vitro* bench-top experiments. A total of 20 combinations with various concentrations of vitamins C and E were prepared to select the best combinations to lower the peroxidation in the AIO-TPN (Supplemental Table 5).

### Combination of copper and zinc

Combinations of zinc at 0.1 mg (10.2 μM), 0.25 mg (25.5 μM), and 0.3 mg (30.6 μM) (standard concentration) and copper at 0.01 mg (1.04 μM), 0.02 mg (2.09 μM), 0.03 mg (3.15 μM), and 0.04 mg (4.20 μM) (standard concentration) for 150 mL AIO-TPN were used for *in vitro* experiments. A total of 12 combinations with various concentrations of zinc and copper were prepared to select the best combinations to lower the peroxidation in AIO-TPN (Supplemental Table 6).

### Preparation of selenium for AIO-TPN

Selenium in the form of selenium dioxide was also added. The baseline concentration of selenium in the AIO-TPN solution was 0.12 μM (2 μg/150 mL). To determine the optimal selenium concentrations for the AIO-TPN, 3 μg (0.18 μM), 4 μg (0.24 μM), 5 μg (0.30 μM), and 6 μg (0.36 μM) were added to selenium-free AIO-TPN.

### Preparation of glutathione for AIO-TPN

Various concentrations of glutathione were added and examined for peroxide concentrations. Glutathione is not generally added to the TPN for neonates [8]. The oxidized form of glutathione (GSSG) was added at the amounts of 1 mg (10 μM), 2 mg (20 μM), 3 mg (30 μM), 4 mg (40 μM), and 5 mg (50 μM) to the 150 mL AIO-TPN.

### Measurement of peroxides using a ferrous oxidation-xylenol orange assay

Peroxides generated in the various TPN solutions were measured using a modified ferrous oxidation-xylenol orange (FOX) assay (version II assay) for lipid emulsions [29], adapted from the FOX version I assay [30]. Briefly, FF-TPN, SMOFlipid, and AIO-TPN were prepared fresh on the day of experiments. The FOX version II reagent was also freshly prepared, consisting of 90% methanol (HPLC grade), 25 mM H_2_SO_4_, 4 mM 99.5% BHT, 250 μM ammonium ferrous sulfate, and 100 μM xylenol orange. TPN samples were diluted at a ratio of 1:1 with Milli-Q ultrapure water, then 50 μL of standards, samples, a positive control (100 μM H_2_O_2_ added TPN samples), and blank (water) were placed into their own 1.5 mL microcentrifuge tubes followed by the addition of 950 μL of FOX-II reagent. After incubation at room temperature for 30 min, absorbance at 560 nm was read using a Synergy Mx 96-well plate reader (BioTek). A standard curve was prepared using 30% H_2_O_2_ solution, which was diluted to 0, 0.1, 0.2, 0.3, 0.4, 0.5, 0.6, and 0.7 mM.

### Statistical analyses

All of the results are expressed as the mean ± SD of ≥3 biological replicates of an experiment with 3 technical replicates of each. Statistical analyses were performed using 1-way Analysis of Variance (ANOVA) followed by multiple comparisons using Tukey’s Honestly Significant Difference (HSD) for pairwise comparisons of categorical data, or Dunnett’s post hoc tests for comparisons of each categorical data with control. Two-factor dependent variables were analyzed using 2-way ANOVA followed by multiple comparisons using Tukey’s HSD. GraphPad Prism (version 8.0.1) was used for analyses and generating graphs. Differences were considered statistically significant when the *P* value was < 0.05.

## Results

### Peroxide concentrations of light-protected and LE TPN samples

**FIGURE 1:**
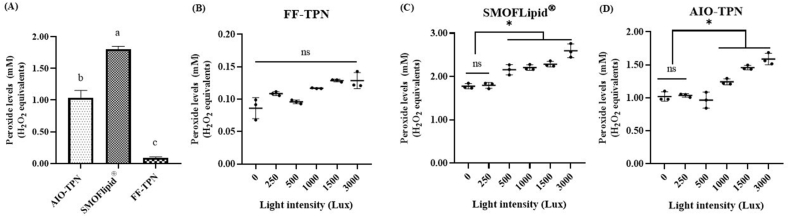
Peroxide concentrations of parenteral nutrition solutions. (A) The peroxide concentrations, expressed as H_2_O_2_ equivalents, were measured from fat-free (FF)-TPN and all-in-one (AIO)-TPN solutions immediately after preparation, and from newly opened bags of SMOFlipid at time zero (0 h). (B–D) The light intensity for TPN solutions, measured in Lux, was adjusted in a dark room to control the external light; ∗*P* < 0.05. Different lowercase letters indicate significant differences (*P* < 0.05) among the peroxide concentrations generated in TPN solutions. Significant differences were determined by 1-way ANOVA followed by multiple comparisons using Tukey’s HSD (A) or Dunnett’s post hoc test (B–D). Bar chart and error bar of column scatter dot plot graph represent the mean ± SD (*n* = 3). TPN, total parenteral nutrition; ANOVA, analysis of variance; HSD, honestly significant difference.

Peroxide concentrations at time zero in the FF-TPN were 0.093 (±0.013) mM H_2_O_2_ equivalents, whereas the peroxide concentrations of a newly opened bag of SMOFlipid were 1.8 (±0.047) mM (*P* < 0.0001) (Figure 1A). The peroxide concentrations significantly elevated to 1.039 (±0.116) mM after adding SMOFlipid at the ratio of 1:11 to the FF-TPN to prepare AIO-TPN (*P* < 0.0001).

To assess the influence of light intensity on peroxide formation, the FF-TPN, SMOFlipid, and AIO-TPN were incubated at 34°C for 24 h at the light exposure of 0, 250, 500, 1000, 1500, or 3000 Lux. Increasing light intensity did not affect the peroxide formation in FF-TPN (*P* > 0.05) (Figure 1B), whereas increasing light intensity influenced the peroxide formation in SMOFlipid and AIO-TPN (Figure 1B and C).

### Peroxide concentrations in light-protected TPN solutions at time zero and after 24 h of dark or light exposure

**FIGURE 2:**
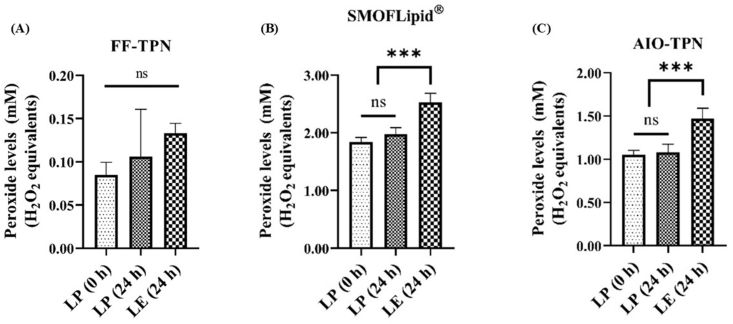
Peroxide concentrations of light-exposed (LE) and light-protected (LP) parenteral nutrition at time zero or for 24 h. (A) Fat-free TPN (FF-TPN) (*n* = 4); (B) SMOFlipid (*n* = 3); (C) All-in-one TPN (AIO-TPN) (*n* = 8). Bars represent the mean ± SD; ∗∗∗*P* < 0.001; significant differences were determined by 1-way ANOVA followed by multiple comparisons using Tukey’s HSD. LP (0 h): light-protected sample at time zero; LP (24 h): light-protected sample, incubated for 24 h; LE (24 h): light-exposed sample for 24 h. TPN, total parenteral nutrition; ANOVA, analysis of variance; HSD, honestly significant difference.

Three aliquots of 2 mL undiluted FF-TPN, SMOFlipid, and AIO-TPN were immediately sealed under nitrogen after preparation. The first aliquot of each solution was wrapped with aluminum foil [light-protected (LP) sample for 24 h] and incubated at 34°C for 24 h. The second aliquot of each solution was incubated at 34°C for 24 h under light exposure of 3000 Lux. The last aliquot of each solution was examined for peroxide concentrations immediately after preparation (at time zero). The formation of peroxide in FF-TPN was not significantly different between LP or LE samples [LP (0 h) = 0.085 (±0.014); LP (24 h) = 0.106 (±0.054); and LE (24 h) = 0.133 (±0.112) H_2_O_2_ equivalents, respectively] (Figure 2A). The peroxide concentrations generated in SMOFlipid and AIO-TPN, exposed to light for 24 h, were significantly increased compared with LP samples at time zero [1.838 (±0.083) compared with 2.526 (±0.159) and 1.050 (±0.055) compared with 1.475 (±0.117) mM H_2_O_2_ equivalents, respectively) (*P* < 0.0001)]. However, peroxide concentrations did not significantly differ between LP at time zero and for 24-h samples in both SMOFlipid and AIO-TPN (Figure 2B and C). This study has revealed that the light exposure to the SMOFlipid or AIO-TPN for 24 h generated a higher amount of peroxides compared with LP samples.

### Role of antioxidants on the peroxide concentrations

#### Vitamin C–added AIO-TPN and peroxide concentrations

**FIGURE 3:**
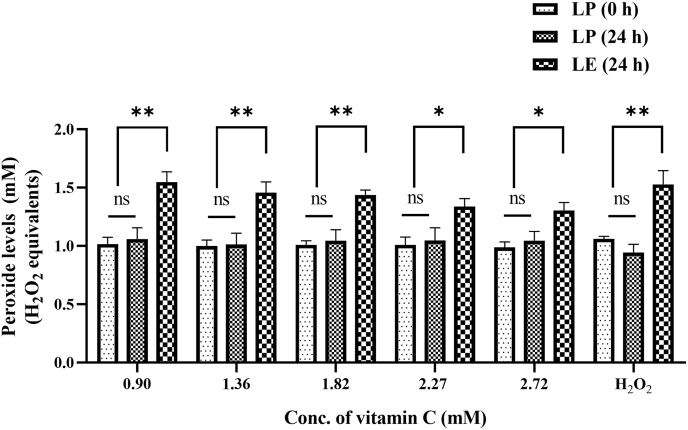
Peroxide concentrations of light-exposed (LE) for 24 h and light-protected (LP) vitamin C–added AIO-TPN at time zero (0 h) or for 24 h (*n* = 3). Vitamin C was added to AIO-TPN to prepare 1.36, 1.82, 2.27, and 2.72 mM concentrations; baseline concentration was 0.9 mM; H_2_O_2_ at 100 μM used as a positive control; Peroxide concentrations are expressed in H_2_O_2_ equivalents. Bars represent the mean ± SD. ∗*P <* 0.05; ∗∗*P <* 0.01. Significant differences were determined by 1-way ANOVA followed by multiple comparisons using Tukey’s HSD. LP (0 h): light-protected sample at time zero; LP (24 h): light-protected sample, incubated for 24 h; LE (24 h): light-exposed sample for 24 h. AIO, all-in-one; TPN, total parenteral nutrition; ANOVA, analysis of variance; HSD, honestly significant difference.

Vitamin C was added to the standard AIO-TPN, prescribed for newborns, at increasing concentrations. The peroxide concentrations of 24-h LE samples were not significantly different with increasing concentrations of vitamin C from 0.90 to 2.27 mM (Figure 3). For instance, the peroxide concentrations observed in LE for 24-h AIO-TPN that had vitamin C at 2.72 mM were 1.304 (±0.097) mM H_2_O_2_ equivalents, compared with that of vitamin C at 0.90 mM concentration [1.547 (±0.125)] (*P* > 0.05) (Figure 3). Even though LP for 24-h AIO-TPN had slightly higher peroxide concentrations than LP AIO-TPN at time zero, it was not significantly different (*P* > 0.05) based on 1-way ANOVA (Figure 3).

#### Vitamin E–added AIO-TPN and peroxide concentrations

**FIGURE 4:**
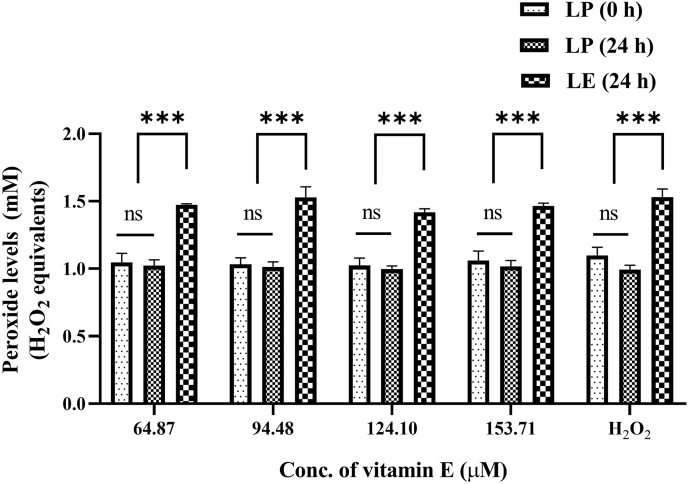
Peroxide concentrations in vitamin E–added light-protected (LP) AIO-TPN at time zero and for 24 h and light-exposed (LE) AIO-TPN for 24 h. AIO-TPN was supplemented with vitamin E from 94.48 to 153.71 μM (the baseline concentration was 64.87 μM). H_2_O_2_ at 100 μM was used as a positive control. peroxide concentrations are expressed in H_2_O_2_ equivalents. Bars represent the mean ± SD (*n* = 3); ∗∗∗*P* < 0.001. Significant differences were determined by 1-way ANOVA followed by multiple comparisons using Tukey’s HSD. LP (0 h): light-protected sample at time zero; LP (24 h): light-protected sample, incubated for 24 h; LE (24 h): light-exposed sample for 24 h. AIO, all-in-one; TPN, total parenteral nutrition; ANOVA, analysis of variance; HSD, honestly significant difference.

Vitamin E (DL-α-tocopherol acetate) was added to the standard AIO-TPN at increasing concentrations. The peroxide concentrations generated in 24-h LE AIO-TPN solutions did not change by increasing the concentration of vitamin E from 64.87 to 152.5 μM [the peroxide concentrations at 64.87 μM = 1.474 (±0.009) mM and at 153.71 μM concentration = 1.466 (±0.026) mM H_2_O_2_ equivalents (*P* > 0.05)] (Figure 4). The peroxide concentrations generated in the LP for 24-h AIO-TPN and LP at time zero samples were not different (*P* > 0.05) (Figure 4) based on 1-way ANOVA.

#### Combined effect of vitamins C and E on peroxidation of AIO-TPN

**FIGURE 5:**
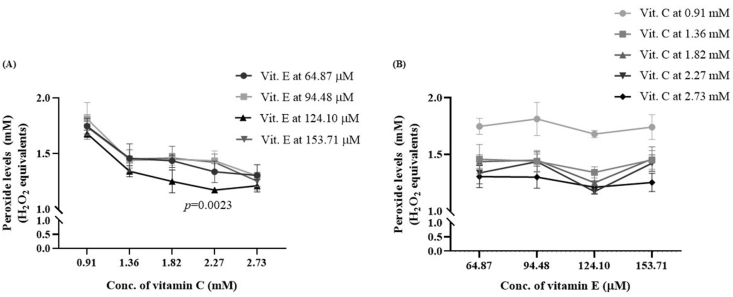
Peroxide concentrations of vitamins C– and E–added AIO-TPN exposed to light for 24 h. AIO-TPN was supplemented with vitamin C from 0.90 to 2.72 mM, and vitamin E from 64.87 to 153.71 μM. H_2_O_2_ at 100 μM was used as a positive control. (A) Increasing concentration of vitamin C with each concentration of vitamin E in the AIO-TPN; (B) increasing concentration of vitamin E with each concentration of vitamin C in the AIO-TPN. Peroxide concentrations are expressed in H_2_O_2_ equivalents. Significant differences were determined by 2-way ANOVA followed by multiple comparisons using Tukey’s HSD. LP (0 h): light-protected sample at time zero; LP (24 h): light-protected sample, incubated for 24 h; LE (24 h): light-exposed sample for 24 h; *n* = 3. AIO, all-in-one; ANOVA, analysis of variance; HSD, honestly significant difference; TPN, total parenteral nutrition.

Vitamins C and E were added at various combinations to AIO-TPN to assess the peroxide concentrations. Increasing combinations of vitamin C and E to the AIO-TPN gradually decreased peroxide concentrations (Figure 5A). When vitamins C and E increased from baseline concentrations (0.90 mM of vitamin C and 0.64 μM of vitamin E) to the combination of vitamin C at 2.27 mM and vitamin E at 124.1 μM, the peroxide concentrations decreased significantly from 1.748 (±0.072) to 1.173 (±0.024) mM H_2_O_2_ equivalents (*P* < 0.0001) (Supplemental Table 7). The highest peroxide concentration [1.873 (±0.146) mM H_2_O_2_ equivalents] was observed in AIO-TPN that had vitamin C at 0.91 mM and vitamin E at 94.48 μM concentrations. Vitamin C at 0.91 mM in all combinations of increasing vitamin E did not decrease the peroxide concentrations (Figure 5B). With vitamin E at 153.7 μM concentrations, the peroxide concentrations were not changed when vitamin C increased from 1.36 to 2.27 mM. In summary, vitamin C at 2.27 mM and vitamin E at 124.1 μM concentrations effectively decreased the peroxide concentrations.

#### Selenium-added AIO-TPN and peroxide concentrations

**FIGURE 6:**
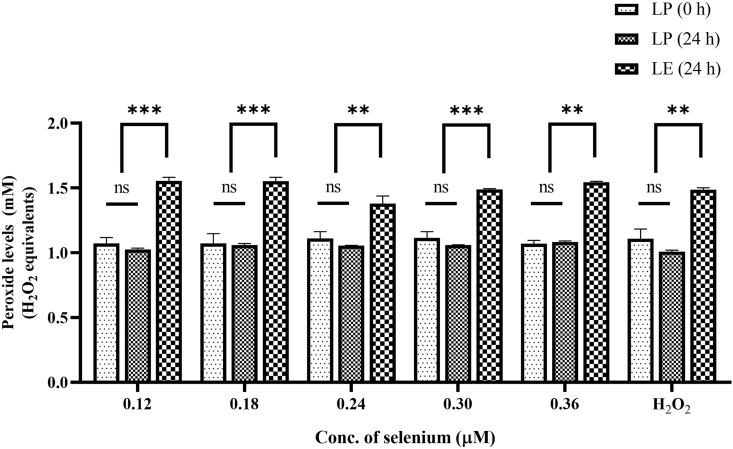
Peroxide concentrations of light-exposed (LE) and light-protected (LP) selenium-added AIO-TPN. AIO-TPN was supplemented with selenium from 0.18 to 0.36 μM (baseline concentration was 0.12 μM). The positive control was H_2_O_2_ at 100 μM. Peroxide concentrations are expressed as H_2_O_2_ equivalents. Bars represent the mean ± SD; *n* = 3. ∗∗∗*P* < 0.001. Significant differences were determined by 1-way ANOVA followed by multiple comparisons using Tukey’s HSD. LP (0 h): light-protected sample at time zero; LP (24 h): light-protected sample, incubated for 24 h; LE (24 h): light-exposed sample for 24 h. AIO, all-in-one; ANOVA, analysis of variance; HSD, honestly significant difference; TPN, total parenteral nutrition.

The selenium concentration of standard AIO-TPN was 0.12 μM (baseline concentration). Increasing the selenium concentrations in AIO-TPN from 0.12 to 0.36 μM, together with light exposure for 24 h, did not significantly change peroxide concentrations [at 0.12 μM concentration =1.554 (±0.039) mM compared with at 0.36 μM = 1.543 (±0.011) mM H_2_O_2_ equivalents]. However, the lowest concentration of peroxide [1.378 (±0.083) mM H_2_O_2_ equivalents] was observed in the standard AIO-TPN, which had the selenium concentration at 0.24 μM concentration (Figure 6).

#### GSSG-added AIO-TPN and peroxide concentrations

**FIGURE 7:**
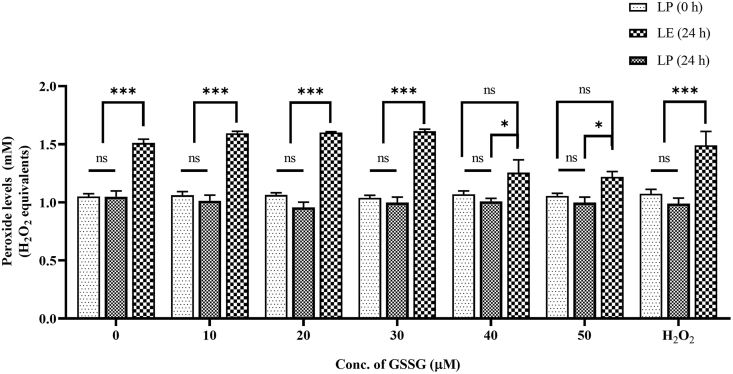
Peroxide concentrations of light-exposed (LE) and light-protected (LP) GSSG-added AIO-TPN. AIO-TPN was supplemented with GSSG from 10 to 50 μM. The positive control H_2_O_2_ was at 100 μM. Peroxide concentrations are expressed in H_2_O_2_ equivalents. Bars represent the mean ± SD (*n*=3); ∗∗∗*P* < 0.001. Significant differences were determined by 1-way ANOVA followed by multiple comparisons using Tukey’s HSD; LP (0 h): light-protected sample at time zero; LP (24 h): light-protected sample, incubated for 24 h; LE (24 h): light-exposed sample for 24 h. AIO, all-in-one; ANOVA, analysis of variance; GSSG, glutathione disulfide; HSD, honestly significant difference; TPN, total parenteral nutrition.

Glutathione, as reduced (GSH) form or as oxidized glutathione disulfide (GSSG), was not included in the commercially available standard AIO-TPN. We added GSSG at increasing concentrations at 0, 10, 20, 30, 40, and 50 μM to the AIO-TPN and incubated for 24 h at 3000 Lux. The peroxide concentrations did not change between 0 and 30 μM. When the GSSG increased from 30 to 40 μM, the peroxide concentrations significantly decreased from 1.612 (± 0.059) to 1.255 (± 0.120) mM H_2_O_2_ equivalents (*P* < 0.01), and it further decreased to 1.218 (± 0.101) mM H_2_O_2_ equivalents at 50 μM concentrations (*P* < 0.001) (Figure 7). Peroxide concentrations within AIO-TPN with 40 μM GSSG were not significantly different between LP at time zero and LE for 24 h. A similar finding was observed for peroxide in AIO-TPN at 50 μM concentrations (Figure 7).

### Combined effect of copper and zinc on peroxide concentrations

**FIGURE 8:**
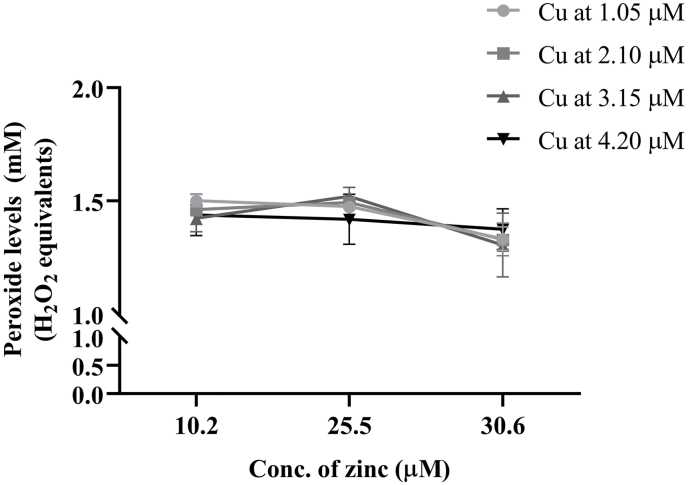
Peroxide concentrations of copper- and zinc-added AIO-TPN exposed to light for 24 h. Copper concentrations in the AIO-TPN were at decreasing concentrations from 4.20 to 1.05 μM and zinc at decreasing concentrations from 30.6 to 10.2 μM. Peroxide concentrations are expressed in H_2_O_2_ equivalents. Significant differences were determined by 2-way ANOVA followed by multiple comparisons using Tukey’s HSD. Two-way ANOVA: copper concentrations on peroxide concentrations, F (3,24) = 0.219, *P* < 0.882; zinc concentrations on peroxide concentrations, F (2, 24) = 12.91, *P* = 0.0002; copper × zinc interaction, F (6, 24) = 0.941, *P* = 0.484. LE (24 h): light-exposed sample for 24 h; *n* = 3. AIO, all-in-one; ANOVA, analysis of variance; HSD, honestly significant difference; TPN, total parenteral nutrition.

Copper and zinc were added at various combinations to AIO-TPN to assess the peroxide concentrations. Decreasing combinations of copper from 4.2 to 1.05 μM and zinc from 30.6 to 10.2 μM did not affect peroxide concentrations (Figure 8; Supplemental Table 8). Two-way ANOVA followed by Turkey’s HSD multiple comparisons revealed that decreasing zinc concentrations increased the peroxide concentrations at fixed copper concentrations [F (2, 24) = 12.91, *P* = 0.0002], whereas decreasing copper concentrations did not decrease the peroxide concentrations at fixed zinc concentrations [F (3, 24) = 0.219, *P* = 0.8822] and no significant interactions found between zinc and copper concentrations [F (6, 24) = 0.9414, *P* = 0.4843].

### Optimal concentration of selected nutrients in the AIO-TPN to minimize the peroxidation

**FIGURE 9:**
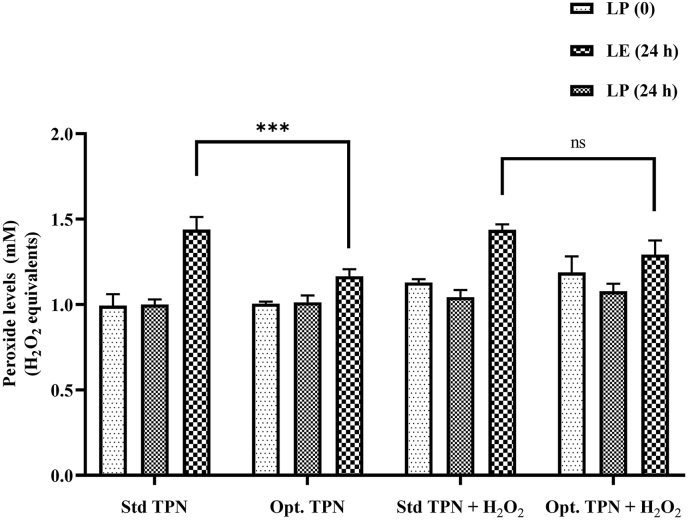
Peroxide concentrations of light-exposed (LE) for 24 h and light-protected (LP) optimized AIO-TPN at time zero or for 24 h. AIO-TPN was supplemented with vitamin C at 2.27 mM, vitamin E at 124.1 μM, selenium at 0.24 μM, oxidized form of glutathione (GSSG) at 40 μM, copper at 4.2 μM, and zinc at 30.6 μM. Peroxide concentrations are expressed in H_2_O_2_ equivalents. Bars represent the mean ± SD (*n* = 3). ∗∗∗*P* < 0.001. Significant differences were determined by 2-way ANOVA: light exposure on peroxide concentrations, F (2,20) = 107.5, *P* < 0.0001; standard and optimized AIO-TPNs on peroxide concentrations, F (3, 20) = 11.06, *P* = 0.0002; light exposure × standard and optimized AIO-TPNs interaction, F (6, 20) = 7.391, *P* = 0.0003. LP (0 h): light-protected AIO-TPN at time zero; LP (24 h): light-protected AIO-TPN, incubated for 24 h; LE (24 h): light-exposed AIO-TPN for 24 h. AIO, all-in-one; ANOVA, analysis of variance; GSSG, glutathione disulfide; HSD, honestly significant difference; TPN, total parenteral nutrition.

An optimized AIO-TPN was formulated with 2.27 mM (equivalent to 60 mg/kg/d) of vitamin C (2.5-fold increment), 88.8 μM (equivalent to 6.3 mg/kg/d) of vitamin E (3-fold increment), 0.24 μM (4 μg/kg/d) of selenium (2-fold increment), 40 μM (equivalent to 4 mg/kg/d) of glutathione (zero concentration at standard TPN), 4.2 μM (equivalent to 40 μg/kg/d) of copper (the same concentration), and 30.6 μM (equivalent to 300 μg/kg/d) of zinc (the same concentration) and had significantly lower peroxide concentrations compared with standard AIO-TPN after 24-h exposure of light [1.165 (±0.042) compared with 1.439 (±0.074) mM H_2_O_2_ equivalents] (*P* < 0.0001) (Figure 9).

## Discussion

Infants born with preterm or gastrointestinal disorders often require TPN. Although this nutrition support is a lifesaving therapy for newborns, several studies have suggested that the administration of TPN causes several complications [[31], [32], [33]]. Oxidants generated in the TPN are believed to cause some of these complications, and can overwhelm the immature endogenous antioxidant systems of newborns [8,34]. The generation of oxidants in TPN can occur through various processes, including nutrient interactions and light exposure. In routine clinical practice, it is often not feasible to shield the hanging bags containing FF-TPN, AIO-TPN, or lipid emulsions, or to shield the tubing from exposure to ambient, day, or phototherapy lighting. Furthermore, stable light intensity in the NICU is challenging. For instance, the light intensity of an NICU is ∼3000 Lux, varying from 600 to 5000 Lux [35,36], depending on the conditions, including phototherapy for icterus neonates. Moreover, light is essential for the circadian function of the newborns, as well as for staff who are on shift work in the NICU. Hence, it is necessary to control the peroxide generation in TPN solutions by controlling other variables than light exposure (3000 Lux).

In this study, newly opened LP SMOFlipid had 1.8 mM H_2_O_2_ equivalent peroxides. Several researchers also found that newly opened parenteral nutrition solutions had significant amounts of peroxides [16,17,37,38]. It is likely that a considerable amount of PUFAs (28.9 % *w/w*) [39] in the SMOFlipid might be oxidized well before reaching the NICU. In routine practice, SMOFlipid is sealed in a transparent bag without appropriate light protection, and the bags are stored in a cardboard box and transported to pharmacy, where they are stored at room temperature.

Light exposure at 3000 Lux resulted in approximately a 40% elevation in peroxide concentrations in AIO-TPN compared with LP counterparts. The elevation of lipid peroxidation could also be explained due to the presence of other components of TPN. For example, riboflavin is a photosensitive vitamin, and is converted to a radical form that attracts hydrogen from PUFAs or other electron donors [8,40,41]. Interestingly, we found that light exposure did not affect peroxide formation of FF-TPN.

Several studies suggest that protecting TPN bags and infusion lines using materials such as aluminum foil, black plastic coverings, or colored tubing (e.g., orange, yellow, or black) can significantly reduce peroxidation. However, simply lowering the light intensity in the NICU or keeping the room dim or dark or covering the bag and lines may impede the detection of critical issues, such as the presence of air bubbles, color changes, or cloudiness in the TPN; disruptions of TPN flow through the line connected to the neonate; and clinical examination of skin and intravenous cannulation. Rather than relying solely on reducing light exposure, a more effective strategy would be to also enhance antioxidant concentrations in TPN solutions to prevent peroxide formation while maintaining safety in the clinical setting.

We showed that increasing vitamin C from 24 to 72 mg/kg/d in TPN did not decrease or increase the peroxide concentrations, which remained high within the range of 1.4–1.5 mM H_2_O_2_ equivalents compared with LP AIO-TPN (1.0 mM H_2_O_2_ equivalents). The lack of effectiveness of the antioxidant properties of vitamin C could be explained by reversible oxidation or prooxidant activity. For instance, vitamin C readily undergoes reversible oxidation to dehydroascorbic acid and further irreversible oxidation to a physiologically inactive form of 2,3-diketogluconic acid, which does not have antioxidant properties [42]. We also showed that increasing vitamin E from 4.6 to 10.9 mg/kg/d to the TPN did not decrease peroxide concentrations. Similarly, King et al. [43] reported that lipid peroxidation was similar between Intralipid and SMOFlipid, regardless of their differing vitamin E content (Intralipid and SMOFlipid have 38 and 200 mg/L of vitamin E, respectively).

The activity of vitamin E depends on other antioxidants, including vitamin C [44]. In this study, combinations of vitamin C at 2.27 mM (equivalent to 60 mg/kg/d) and E at 124.1 μM (equivalent to 8.8 mg/kg/d) significantly decreased the peroxide concentrations from 1.8 to 1.2 mM H_2_O_2_ equivalents. This could be due to the additive effect of vitamin C and E on peroxide concentrations [45]. For instance, α-tocopherol reacts with peroxyl radicals and generates lipid hydroperoxide and the α-tocopheryl radical (Vit E-O^•^). The Vit E-O^•^ accepts the proton from a donor, particularly from vitamin C to form reduced α-tocopherol, and then the latter turns into oxidized vitamin C (dehydroascorbic acid) [46]. If there is a lack of vitamin C or other antioxidants, Vit E-O^•^ can re-initiate lipid peroxidation [47]. Our finding that supplementation of vitamin C (≤60 mg/kg/d) and E (≤8.8 mg/kg/d) for TPN under physiologically safe levels would decrease the peroxide concentrations rather than supplementing vitamin C or vitamin E separately. Previous studies reported that parenteral vitamin C at 75 mg/d (which can increase ≤200 mg/d without adverse effects) can be given [48,49]. Vitamin E is more problematic, as toxicity can occur if the plasma vitamin E concentration reaches to 80 μM [50]. Brion et al. [51] found that vitamin E intravenously provided at high doses of 7 mg/kg/d is safe for very-low birth weight babies.

Although the American Society for Parenteral and Enteral Nutrition (ASPEN) recommends administering 2 μg/kg/d of selenium to newborns [52], numerous clinical studies have reported that a substantial proportion of neonates on parenteral nutrition developed selenium deficiency, characterized by plasma concentrations falling <50 μg/L [[53], [54], [55], [56]]. This suggests that the current recommendations may be insufficient for preventing deficiency or benefit to the neonates, especially in vulnerable populations like preterm infants or those with prolonged dependence on parenteral nutrition [55]. In our study, a slight decline in peroxide concentrations with 0.24 μM (equivalent to 4 μg/kg/d) concentration was observed for standard AIO-TPN, but not with other concentrations; this discrepancy could be due to the interaction with other trace elements, including copper at current recommended concentrations in the standard AIO-TPN [57]. *In vivo*, selenium acts as a cofactor for glutathione peroxidase, which maintains the redox potential of GSH/GSSG [58]. However, as these interactions occur *in vivo*, the redox activity of selenium in the TPN bag is still unclear. Lee et al. [59] suggested increasing the intravenous selenium dose to 4.0 μg/kg/d to maintain the serum selenium concentration above the threshold of 50 μg/L. Additionally, others have found that infusing selenium at the rate of 7 μg/kg/d could be beneficial for neonates [59,60]. Therefore, this study confirms the beneficial effect of adding more selenium with other antioxidants to decrease peroxidation in the TPN solution, and to increase plasma concentrations.

To date, no clinical studies have examined TPN fortified with glutathione for neonates. With our study, supplementation of GSSG at a concentration of 40 μM (equivalent to 4 mg/kg/d) resulted in a significant reduction in peroxide concentrations. Adding glutathione helps to reduce H_2_O_2_ to H_2_O, but this reaction is slow nonenzymatically. Although there is still no definitive consensus on using glutathione in TPN solutions, several studies have suggested the potential benefits of adding glutathione, particularly for premature infants, to enhance antioxidant support and improve outcomes [61]. Hence, the antioxidant status of GSH or GSSG or its stability for TPN should be further examined and optimized by adding the optimal ratio of GSSG:GSH for TPN.

Copper or zinc at higher concentrations act as a prooxidant because of its redox cycling between Cu^2+^ and Cu^+^ [[62], [63], [64]]. In this study, the combination of various concentrations of copper or zinc did not decrease peroxide concentrations. Therefore, lowering or increasing copper or zinc concentrations may not be advantageous for TPN, in terms of peroxidation.

It should be mentioned that in the NICU, TPN administration is gradually decreased as enteral feeding is advanced, according to the neonates’ tolerance. Because antioxidant components are often maintained as fixed daily doses, our findings suggest that their in-bag concentrations should be adjusted based on the combined parenteral and enteral intake to minimize the overall oxidant load.

This study has some potential limitations. Given the large number of ingredients in AIO-TPN, experimentally testing every possible combination is challenging. For example, riboflavin and manganese also impact the peroxide formation and interact with other ingredients; these are for future examination. Moreover, aluminum contamination, which is common in parenteral nutrition products [65], and its associated oxidative stress could be further investigated. Additionally, the stability of added vitamins C and E and glutathione in TPN requires further investigation, as many of these nutrients can degrade during formulation and storage. Furthermore, this study used only one light source to standardize the Lux intensity and explored select nutrients most likely to be affected by oxidation. Examining the additional light sources commonly used in NICUs (such as amber, soft white, cool white, and daylight) could provide a more comprehensive understanding of light-induced peroxide generation. Moreover, different bag materials [ethylene-vinyl acetate (EVA), polyvinyl chloride (PVC), di(2-ethylhexyl) phthalate, or polypropylene-based film], thickness, or use of multilayered bags may impact light penetration and contribute the peroxide generation. Additionally, more specific oxidative products, such as 4-hydroxy-2-nonenal and 4-hydroxy-2-hexanal, could provide further understanding of oxidation in TPN. The FOX-II assay measures total hydroperoxides, without distinguishing between hydrogen peroxide, lipid hydroperoxides, and other organic peroxides. The peroxides derived from different precursors (e.g., ascorbyl or lipid peroxides) may exhibit different oxidative potentials and biological effects *in vivo*. Moreover, *in vitro* optimization focuses primarily on measuring peroxide concentrations, and the biological effects of these changes should be evaluated in cell culture or animal models before translation to clinical settings.

There are also some clinical limitations. We selected SMOFlipid in this *in vitro* study, even though various lipid emulsions are used in different NICUs, because of its proven efficacy in reducing parenteral nutrition (PN)–associated liver disease. However, its impact on oxidative stress has not been extensively studied [8]. Moreover, in routine NICU practice, fat-free PN and intravenous lipid emulsions are often administered separately through a Y-connection, allowing a shorter infusion pathway to the neonate. This approach may generate fewer oxidants because of the shorter exposure time. However, in our study, we used an AIO-TPN admixture (which is increasingly recommended in clinics) as this scenario would provide maximum oxidation potential, future studies could assess peroxidation when FF-TPN and lipid are mixed for a shorter duration as in Y-connection setups.

In conclusion, overall, we developed an optimized AIO-TPN that significantly decreased peroxide content compared with standard AIO-TPN. All of the ingredients added to the optimized AIO-TPN were within the physiological range. This is also the first study that optimized several antioxidants together, including glutathione, in *in vitro* experiments. We showed that external factors appear to account for the bulk of peroxide generation in TPN. Therefore, this study suggests minimizing the light exposure at NICUs or modifying environmental factors by protecting the TPN bag/tubing from light to help reduce peroxides. In the future, we anticipate testing our modified AIO-TPN using cellular and *in vivo* models, to examine whether it reduces cellular stress and oxidant production compared with conventional AIO-TPN. Data from such work will guide us on the efficacy of the modified AIO-TPN for use in newborns.

## Author contributions

The authors’ responsibilities were as follows – KK, RFB, RJB: conceptualization; KK: methodology; KK: software; KK, RFB, RJB: validation; KK: formal analysis; KK: investigation; KK: resources; KK: data curation; KK: writing—original draft preparation; RFB, RJB: writing—review and editing; RFB, RJB: supervision; RFB, RJB: project administration; KK, RFB, RJB: funding acquisition; and all authors: read and agreed to the published version of the manuscript.

## Declaration of generative AI and AI-assisted technologies in the writing process

The authors declare that no generative AI or AI-assisted technologies were used in the writing of this manuscript.

## Data availability

Data described in the manuscript will be made available upon reasonable request to the corresponding authors.

## Funding

This research was funded by Accelerating Higher Education Expansion and Development (Colombo, Sri Lanka) (KK) and the Janeway Research Foundation (St. John’s, NL, Canada) (RFB, RJB).

## Conflict of interest

The authors report no conflicts of interest.
