# Supplementary material for: Antioxidant Supplementation Reduces In Vitro Oxidant Generation in Neonatal Total Parenteral Nutrition Solutions
Source: Curr Dev Nutr. 2026 Apr 15;10(5):107694. doi: 10.1016/j.cdnut.2026.107694 (PMC13194635; doi:10.1016/j.cdnut.2026.107694)
Supplement: multimedia component 1 [file mmc1.docx]

**Appendix A. Supplementary data**

**Table 1.** Amino acid composition for TPN. Primene 10% (*w/v*) is an injectable amino acid solution specifically designed for infants under one year of age. It is a product of Baxter Corporation, Canada. The dosage was determined based on the per-kilogram-per-day requirement for newborns.

| **Amino acids** | **Primene^®^ (Baxter) 10% *w/v*** | **g/kg/day at 150 mL TPN/kg/day** |
| --- | --- | --- |
| ***Indispensable amino acids (g/100 mL)*** |  |  |
| Isoleucine | 0.67 | 0.0536 |
| Leucine | 1.0 | 0.08 |
| Valine | 0.76 | 0.0608 |
| Phenylalanine | 0.42 | 0.0336 |
| Tryptophan | 0.2 | 0.016 |
| Methionine | 0.24 | 0.0192 |
| Lysine | 1.1 | 0.088 |
| Threonine | 0.37 | 0.0296 |
| Histidine | 0.38 | 0.0304 |
| ***Dispensable amino acids*** |  |  |
| Alanine | 0.8 | 0.064 |
| Aspartate | 0.6 | 0.048 |
| Glutamate | 1.0 | 0.08 |
| Serine | 0.4 | 0.032 |
| ***Conditionally indispensable amino acids*** |  |  |
| Cysteine | 0.189 | 0.01512 |
| Tyrosine | 0.045 | 0.0036 |
| Glycine | 0.4 | 0.032 |
| Arginine | 0.84 | 0.0672 |
| Proline | 0.3 | 0.024 |
| Asparagine | - | - |
| Glutamine | - | - |
| ***Non-protein amino acids*** |  |  |
| Ornithine hydrochloride | 0.318 | 0.02544 |
| Taurine | 0.060 | 0.0048 |

**Table 2**: Trace elements (TEs) added to the TPN. Micro+6 contains six TEs for pediatric TPN.

|  | **Micro+6**  **SANDOZ Canada Incorporated** | **Per kg/day at 150 mL TPN/kg/day** |
| --- | --- | --- |
| Zinc sulfate | 3 mg/mL | 0.3 mg |
| Copper sulfate | 0.4 mg/mL | 0.04 mg |
| Manganese sulfate | 0.1 mg/ml | 0.01 mg |
| Chromic chloride | 4 μg/mL | 0.4 μg |
| Selenious acid | 20 μg/mL | 2 μg |
| Sodium iodine | 60 μg/mL | 6 μg |

**Table 3**: Electrolytes for TPN with manufacturer name and dose rate for newborns.

| **Electrolytes** | **Manufacturer** | **Per kg/day at 150 mL TPN/kg/day** |
| --- | --- | --- |
| Magnesium sulfate injection USP 20% (200 mg/mL or 0.8 mmol/mL) | SANDOZ, Canada | 0.2 mmol |
| Calcium gluconate injection, USP 10% (100 mg/mL or 0.465 mEq/mL/0.232 mmol/mL | Fresenius Kabi, Canada | 0.46 mmol |
| Potassium phosphate injection, USP (15 mL)   - Phosphorus (3 mmol/mL) - Potassium (4.4 mEq/mL) | Fresenius Kabi, Canada | 4.08 mmol  5.98 mEq |
| Sodium acetate injection, USP 32.8% (4 mmol/mL or 4 mEq) anhydrous | Omega, Canada | 3 mmol |
| Sodium chloride injection, USP 23.4% (4 mmol/mL) | Pfizer, Canada | 2 mmol |

**Table 4**: Composition of vitamins for parenteral nutrition. Multi-12/K_1_^®^ pediatric multiple vitamins for infusion were used.

| **Vitamins** | **Multi-12/K1 Pediatric** | **Per kg/day at 150 mL TPN/kg/day** |
| --- | --- | --- |
| ***Vial 1 (4 mL)*** |  |  |
| Ascorbic acid | 80 mg | 24 mg |
| Vitamin A | 2300 IU | 690 IU |
| Vitamin D | 400 IU | 120 IU |
| Thiamine (as hydrochloride) | 1.2 mg | 0.36 mg |
| Riboflavin (as phosphate) | 1.4 mg | 0.42 mg |
| Pyridoxine hydrochloride | 1 mg | 0.3 mg |
| Niacinamide | 17 mg | 5.1 mg |
| *d*-Panthenol | 5 mg | 1.5 mg |
| Vitamin E (DL-alpha tocopherol acetate) | 7 IU | 2.1 IU |
| Vitamin K_1_ | 0.2 mg | 0.06 mg |
| ***Vial 2 (1 mL)*** |  |  |
| Biotin | 20 μg | 6 μg |
| Folic acid | 140 μg | 42 μg |
| Vitamin B12 (cyanocobalamin) | 1 μg | 0.3 μg |
| ***Other ingredients*** |  |  |
| Polysorbate 80 | 1.25% | - |
| Sodium hydroxide or HCl or Sodium citrate or citric acid | To adjust pH | - |
| Mannitol | 7.5% | - |

**Table 5: Combinations with various concentrations of vitamins C and E.** Vitamin E at 4 concentrations and vitamin C at 5 concentrations were prepared and combined. Concentrations A, B, C, D, and E are 0.91, 1.36, 1.81, 2.37, and 2.73 μM of vitamin C, respectively. Concentrations of a, b, c, and d are 64.87, 94.48, 124.10, and 153.7 μM of vitamin E, respectively.

|  |  | **Vitamin E**  **mg (μM)** | | | |
| --- | --- | --- | --- | --- | --- |
|  |  | 4.6 (64.87)*  (Conc. a) | 6.7 (94.48)  (Conc. b) | 8.8 (124.10)  (Conc. c) | 10.9 (153.7)  (Conc. d) |
| **Vitamin C mg/μM** | 24 (0.91)*  (Conc. A) | Aa | Ab | Ac | Ad |
|  | 36 (1.36)  (Conc. B) | Ba | Bb | Bc | Bd |
|  | 48 (1.81)  (Conc. C) | Ca | Cb | Cc | Cd |
|  | 60 (2.37)  (Conc. D) | Da | Db | Dc | Dd |
|  | 72 (2.73)  (Conc. E) | Ea | Eb | Ec | Ed |

*Baseline concentration found in the AIO-TPN (standard) for newborns.

**Table 6:** **Combinations with various concentrations of zinc and copper.** Zinc at 3 concentrations and copper at 4 concentrations were prepared and combined. Concentrations A, B, and C are 10.2, 25.5, and 30.6 μM of zinc, respectively. Concentrations of a, b, c, and d are 1.04, 2.09, 3.15, and 4.20 μM of copper, respectively.

|  |  | **Copper**  **mg (μM)** | | | |
| --- | --- | --- | --- | --- | --- |
|  |  | 0.01 (1.04)  (Conc. a) | 0.02 (2.09)  (Conc. b) | 0.03 (3.15)  (Conc. c) | 0.04 (4.20)*  (Conc. d) |
| **Zinc**  **mg (μM)** | 0.1 (10.2)  (Conc. A) | Aa | Ab | Ac | Ad |
|  | 0.25 (25.5)  (Conc. B) | Ba | Bb | Bc | Bd |
|  | 0.3 (30.6)*  (Conc. C) | Ca | Cb | Cc | Cd |

*Baseline concentration found in the AIO-TPN (standard) for newborns

**Table 7. Peroxide levels in AIO-TPN after adding various concentrations of vitamins C and E.** The vitamin C at the concentrations of 24 mg (0.91 mM) (baseline), 36 mg (1.36 mM), 48 mg (1.82 mM), 60 mg (2.27 mM), and 72 mg (2.73 mM) per day; and vitamin E at 4.6 mg (64.87 μM) (baseline), 6.7 mg (94.48 μM), 8.8 mg (124.10 μM), and 10.9 mg (153.7 μM) per day; were added.

|  |  | **Vitamin E**  **mg (μM)** | | | |
| --- | --- | --- | --- | --- | --- |
|  |  | **4.6 (64.87)***  **Mean ± SD** | **6.7 (94.48)**  **Mean ± SD** | **8.8 (124.10)**  **Mean ± SD** | **10.9 (153.71)**  **Mean ± SD** |
| **Vitamin C**  **mg (μM)** | **24 (0.91)*** | 1.748 (0.072) | 1.813 (0.146) | 1.679 (0.032) | 1.74 (0.110) |
|  | **36 (1.36)** | 1.458 (0.129) | 1.440 (0.095) | 1.342 (0.049) | 1.452 (0.082) |
|  | **48 (1.82)** | 1.436 (0.060) | 1.450 (0.054) | 1.250 (0.102) | 1.460 (0.107) |
|  | **60 (2.27)** | 1.337 (0.097) | 1.436 (0.087) | 1.173 (0.024) | 1.422 (0.074) |
|  | **72 (2.73)** | 1.304 (0.097) | 1.300 (0.098) | 1.212 (0.056) | 1.253 (0.080) |

*Baseline concentration found in the AIO-TPN (standard) for newborns. Two-way ANOVA was performed to analyze the combination effect of vitamins C and E on the generation of peroxide levels. The main effect analysis of vitamin C depicted a statistically significant effect on decreasing the peroxide levels (F (4, 40) = 52.09, *p* < 0.0001). The main effect analysis of vitamin E showed a statistically significant effect on decreasing the peroxide levels (F (3, 40) = 9.646, *p* < 0.0001). The results showed that there was no statistically significant interaction between the effects of vitamins C and E (F (12, 40) = 0.734, *p* = 0.711).

**Table 8: Peroxide levels in AIO-TPN after adding various concentrations of copper and zinc.** Zinc at 0.1 mg (10.2 μM), 0.25 mg (25.5 μM), and 0.3 mg (30.6 μM) (baseline concentration); and copper at 0.01 mg (1.04 μM), 0.02 mg (2.09 μM), 0.03 mg (3.15 μM), and 0.04 mg (4.20 μM) (baseline concentration); were added.

|  |  | **Copper**  **mg (μM)** | | | |
| --- | --- | --- | --- | --- | --- |
|  |  | - 1. **(1.04)**   2. **Mean ± SD** | - 1. **(2.09)**   2. **Mean ± SD** | **0.03 (3.15)**  **Mean ± SD** | - 1. **(4.20)***   2. **Mean ± SD** |
| **Zinc**  **mg (μM)** | **0.1 (10.2)** | 1.501 (0.027) | 1.463 (0.026) | 1.424 (0.061) | 1.439 (0.092) |
|  | **0.25 (25.5)** | 1.475 (0.026) | 1.494 (0.024) | 1.520 (0.040) | 1.420 (0.111) |
|  | **0.3 (30.6)*** | 1.334 (0.052) | 1.330 (0.072) | 1.305 (0.141) | 1.376 (0.091) |

*Baseline concentration found in the AIO-TPN (standard) for newborns. Two-way ANOVA was performed to analyze the combination effect of copper and zinc on the generation of peroxide levels. The main effects analysis of copper depicted no significant effect on decreasing the peroxide levels (F (3, 24) = 0.219, *p* = 0.8822). The main effects analysis of decreasing zinc showed a statistically significant effect on increasing the peroxide levels (F (2, 24) = 12.91, *p* = 0.0002). The results showed that there was no significant interaction between the effects of copper and zinc (F (6, 24) = 0.941, *p* = 0.484).
